# Assessment and Comparison of Phenomenological and Physical Constitutive Models for Predicting the Hot Deformation Behavior of Metallic Materials: A Pathway for Sustainable Metal Forming in Al-Kharj Governorate

**DOI:** 10.3390/ma18092061

**Published:** 2025-04-30

**Authors:** Ali Abd El-Aty, Abdallah Shokry

**Affiliations:** 1 Department of Mechanical Engineering, College of Engineering at Al Kharj, Prince Sattam Bin Abdulaziz University, Al Kharj 11942, Saudi Arabia; 2 Department of Mechanical Engineering, Faculty of Engineering, Fayoum University, Fayoum 63514, Egypt

**Keywords:** Al-Kharj, hot deformation, constitutive modeling, Johnson–Cook, Fields and Backofen, Khan–Huang–Liang, Zerilli–Armstrong

## Abstract

In the context of Al-Kharj city, which is steadily advancing as an industrial and manufacturing hub within Saudi Arabia, this study has significant relevance. The city’s focus on metal forming, fabrication, and materials engineering makes it crucial to optimize processes such as hot deformation of metallic alloys for various sectors, including aerospace, automotive, oil and gas, and structural applications. By assessing and comparing phenomenological and physical material models for nickel, aluminum, titanium, and iron-based alloys, this study aids Al-Kharj industries in advancing their process simulation and predictive performance. Thus, this study aims to evaluate the proposed phenomenological and physically based constitutive models for Ni-, Al-, Ti-, and Fe-based alloys to enhance the accuracy of high-temperature deformation simulations. Phenomenological models investigated include the Johnson–Cook (JC), Fields and Backofen (FB), and Khan–Huang–Liang (KHL) formulations, while the Zerilli–Armstrong (ZA) model represents the physical category. Additionally, various modifications to these models are explored. Model parameters are calibrated using the Levenberg–Marquardt algorithm to minimize mean square error. Performance is assessed through key statistical metrics, including the correlation coefficient (R), average absolute relative error (AARE), and root mean square error (RMSE). Of the 32 models analyzed, a modified version of the JC model delivers the highest accuracy across all alloys. Furthermore, four other modifications, one each for the JC and ZA models and two for the FB model, exhibit superior predictive capability for specific alloys. This makes this study valuable not just academically, but also as a practical resource to boost Al-Kharj’s industrial competitiveness and innovation capacity.

## 1. Introduction

Hot deformation involves the high-temperature shaping of metallic materials under conditions where dynamic recrystallization dominates. This process enables the formation of different microstructures and mechanical properties through recrystallization [1,2]. High-temperature metalworking operations, notably thermal rolling, forging, and direct extrusion, are standard industrial practices for alloy forming. These processes can be influenced by factors such as strain hardening, dynamic recovery, and softening, which may impact the forming behavior of the alloys [3,4,5]. Thus, optimizing the hot deformation parameters can enhance the performance of the alloy. Beyond hot-forming processes, numerous alloys are selected for high-temperature applications because of their exceptional strength and thermal stability. Nickel-based superalloys, particularly those with high Cr content, are extensively used in industries such as aerospace, energy, and nuclear power due to their exceptional mechanical strength, creep resistance, and corrosion stability at elevated temperatures [6]. These alloys are engineered to maintain structural integrity under extreme thermal and mechanical loads, making them indispensable in components like turbine blades, combustion chambers, and reactor cores [7]. For instance, Ni-Cr superalloys serve as critical materials in gas turbines and nuclear reactors, where thermal stability and resistance to oxidation are paramount. Similarly, Ti alloys are widely employed in aerospace structural components owing to their high strength-to-weight ratio and excellent fatigue resistance [6,7,8]. Beyond these, materials such as AA7020 Al alloys are preferred in airframe structures subjected to dynamic loading, while FeCr alloys are commonly used in high-speed rotating machinery due to their strength and wear resistance under severe operating conditions [9,10]. Consequently, accurately predicting the flow behavior of these alloys during hot deformation and under dynamic loading conditions is essential for optimizing manufacturing processes and ensuring component reliability in demanding environments [11,12,13].

Constitutive equations are integral to finite element simulations of hot deformation processes. These simulations aid in optimizing process parameters and predicting potential issues before actual production. Finite element software incorporates various models to simulate and replicate hot working processes and real-world applications, such as the JC model [14,15,16] and the ZA model [17,18]. Analytical approaches for characterizing the mechanical response of materials across different processing conditions typically fall into three groups: phenomenological formulations that use experimentally derived relationships to capture deformation characteristics [19,20,21,22,23], physical material relationships that incorporate fundamental material physics [24,25,26], and intelligence-based models, which utilize machine learning techniques to predict flow stress [27,28,29,30]. Selecting the appropriate constitutive model is essential for accurately predicting flow behavior during hot deformation and optimizing manufacturing processes. This is because the process involves complex interactions among material properties, temperature, strain rate, and microstructural evolution.

The Johnson–Cook (JC) formulation, developed in 1983, remains a benchmark empirical approach for modeling thermomechanical material response under varying process conditions [31]. The JC formulation represents a widely implemented solution in both academic research and industrial applications involving material behavior prediction. The JC formulation integrates three key mechanisms to represent material response under severe loading: work hardening, rate sensitivity, and temperature-induced softening. While effective, the conventional JC framework neglects interactions among critical thermomechanical variables—including deformation level, loading rate, and thermal effects—leading to potential inaccuracies in flow stress estimation, especially for advanced alloys under complex processing conditions. To overcome these limitations, multiple studies have introduced refined versions of the JC model, improving its reliability for diverse material systems and operational scenarios [32,33,34,35,36,37,38,39,40,41,42,43,44,45,46,47,48]. These modifications include the introduction of additional terms, parameters, or functions to account for the interactions and dependencies between the governing factors, providing more accurate and robust descriptions of material behavior. One of the key strengths of the JC model is its seamless implementation in finite element software, enabling the simulation of material behavior under hot working and real application conditions.

Among empirical constitutive frameworks, the Fields–Backofen (FB) formulation serves as a benchmark for characterizing elevated-temperature plastic response during thermomechanical processing [49]. Despite its effectiveness, the FB model has a notable limitation: it does not explicitly account for thermal softening, a key factor influencing material flow at elevated temperatures. This omission means that while the model provides accurate predictions for certain alloys and material systems [50,51], its reliability diminishes for others, particularly those significantly affected by thermal softening [52,53]. To overcome this limitation, multiple studies have developed refined versions of the FB approach. These adaptations incorporate thermal softening mechanisms into the constitutive framework, enhancing prediction reliability for diverse alloy systems and manufacturing scenarios [54,55,56,57,58].

The Khan–Huang–Liang (KHL) model is a widely recognized phenomenological approach for predicting the hot flow behavior of materials, particularly under dynamic loading conditions [59]. A key advantage of this approach is its consideration of the coupled effects of dislocation accumulation and annihilation mechanisms, which dominate the plastic response of engineering materials subjected to hot working operations. Despite its versatility and broad applicability, the accuracy of the KHL model can be limited for certain alloys, especially those exhibiting complex deformation behaviors or pronounced sensitivity to microstructural changes. Nevertheless, it has been shown to provide reliable predictions for a wide range of materials, making it a valuable tool in material science and engineering [60,61]. To address its limitations and enhance its predictive capability, researchers have proposed various modifications to the KHL model [62,63,64].

Among microstructure-sensitive formulations, the Zerilli–Armstrong (ZA) framework has gained prominence as a physics-grounded approach for characterizing thermomechanical material response [65]. Its strength lies in quantifying the coupled effects of mechanical and thermal variables across extensive processing parameter ranges. Nevertheless, the conventional ZA approach has notable shortcomings, particularly its failure to incorporate key material response phenomena, including dislocation accumulation, thermal recovery processes, and grain structure transformations. This omission restricts its capability to fully capture the complex flow behavior observed in materials during high-temperature deformation. As a result, multiple research groups have advanced refined iterations of the base formulation, significantly expanding its precision and practical utility [66,67,68,69]. These enhancements typically involve incorporating additional parameters or reformulating the model equations to better reflect the influence of microstructural changes and deformation mechanisms, making the ZA model more robust for practical applications in material processing and design. The ZA model is also integrated into finite element software, allowing the simulation of material behavior during hot working and under real-world application conditions.

The Levenberg–Marquardt optimization scheme serves as a key computational tool for identifying material parameters in constitutive relations that characterize high-temperature forming behavior. By merging the stability of gradient-based search with the efficiency of second-order approximation techniques, this approach achieves reliable solutions for challenging nonlinear systems [70]. In the context of hot deformation, where the flow stress is influenced by temperature, strain, and strain rate, accurate parameter identification is essential to capture the material’s response under various conditions. The Levenberg–Marquardt algorithm efficiently minimizes the error between experimental data and model predictions, ensuring precise calibration of constitutive models. This, in turn, enhances the reliability of simulations used in designing and optimizing industrial metal forming processes, reducing experimental costs and improving product quality.

This study examines and evaluates a range of phenomenological and physical models used to predict the hot deformation behavior of various element-based alloys, including those based on nickel, aluminum, titanium, and iron. The Levenberg–Marquardt algorithm is employed to optimize model parameters, with the goal of minimizing the mean squared error. The analysis utilizes established statistical metrics such as the correlation coefficient (R), average absolute relative error (AARE), and root mean square error (RMSE).

## 2. Experimentation

This study focuses on four distinct categories of alloys, each with a different base element, to analyze the associated phenomenological and physical models. The hot deformation behavior of these alloys was previously investigated and reported in various studies: U720LI, a Ni-based alloy, in [71]; AA7020, an Al-based alloy, in [72]; Ti-4.5Al-1V-3Fe, a Ti-based alloy, in [73]; and 40Cr steel, an Fe-based alloy, in [74]. The stress–strain experimental data utilized in this work were extracted from the respective publications using the open-source software Plot Digitizer 2.6.11b, encompassing data for all four alloys under high-temperature conditions and varying strain rates. A statistically based study was conducted to identify the data extraction accuracy of the software utilized. The data chart with the highest nonlinearity was chosen and scanned. The data sets were then extracted three times using the Plot Digitizer software. Statistical assessments demonstrated maximum prediction variances of 1.019 × 10^−6^ (strain) and 4.31 MPa (stress), with relative discrepancies not exceeding −0.25% and 0.58%, respectively. The experimental data are selected to cover a wide range of deformation conditions, including high temperatures and various strain rates, which ensures that the models are well-calibrated. This study examined four distinct alloy systems—nickel-based U720LI, aluminum alloy AA7020, titanium alloy Ti-4.5Al-1V-3Fe, and chromium steel 40Cr—under controlled thermomechanical conditions. Strain rates of 0.001–1 s^−1^ were applied to the first three materials at respective temperature intervals of 1040–1160 °C, 400–520 °C, and 750–950 °C, while the steel was evaluated at 0.01–10 s^−1^ between 750 and 1050 °C. All materials exhibited consistent deformation trends: elevated strain rates enhanced stress resistance through dislocation multiplication, whereas higher temperatures promoted stress relaxation via thermal activation. The characteristic stress evolution progressed through three phases: (1) rapid initial hardening during early deformation, (2) progressive softening through dynamic recovery, and (3) equilibrium stabilization when hardening and softening mechanisms reached parity.

## 3. Constitutive Models

High-temperature material models establish functional relationships capturing the combined impact of mechanical stresses, plastic strains, thermal environment, and deformation rate in manufacturing processes including forging, rolling, and extrusion. These models incorporate material-specific parameters, such as activation energy, flow stress, and microstructural evolution, to capture the effects of deformation mechanisms like dislocation motion, grain boundary sliding, and phase transformation. Accurate constitutive models are crucial for optimizing processing parameters and improving the performance and quality of materials in high-temperature applications.

The following analysis explores major categories of material response models, encompassing both phenomenological and physics-based approaches such as the classical JC, FB, KHL, and ZA formulations, while emphasizing their significant methodological enhancements.

### 3.1. Original Johnson–Cook Model

The JC model [31], established in 1983, remains a benchmark for simulating deformation characteristics in metals subjected to various processing conditions. The formulation decomposes material responses into three independent contributions: dislocation accumulation, loading rate effects, and heat-induced softening. Notably simple in its structure, the model requires only five constants for its application and is expressed as follows:

(1) $$\sigma =\left(A+B\varepsilon^{n}\right)\left(1+C\ln\varepsilon^{\cdot *}\right)\left(1-{T^{*}}^{m}\right)$$

The variable σ captures the instantaneous deformation resistance, while ε measures the progressive plastic distortion. Among the model parameters, A reflects the baseline yield strength, the pair (B, n) governs strain hardening characteristics (as scaling factor and nonlinearity parameter), and (C, m) controls strain–rate dependence and thermal degradation of strength. The dimensionless strain rate $\varepsilon^{\cdot *}$ is obtained by normalizing the current strain rate $\varepsilon^{\cdot }$ against a selected reference value $\varepsilon_{{}^{\circ}}^{\cdot }$. The temperature parameter $T^{*}$ represents a normalized thermal state, calculated using the current temperature T offset by reference temperature $T_{r}$ and scaled by the difference between melting point $T_{m}$ and $T_{r}$.

The Johnson–Cook formulation has become a standard framework in computational material science for characterizing deformation response across diverse loading scenarios, but it has notable limitations during hot deformation. The model assumes a decoupled influence of strain, strain rate, and temperature, which can lead to inaccuracies in representing dynamic recrystallization, grain growth, or phase transformations that are critical during hot deformation. This limitation makes the JC model less reliable for processes involving complex thermomechanical conditions, such as hot rolling or forging, where more advanced models incorporating microstructural evolution are often preferred. Therefore, several modifications were presented.

#### 3.1.1. Meyers’s Revised JC

The baseline JC model was advanced by Meyers et al. [34] via implementation of an exponential thermal term, replacing the initial softening expression. The modified form often better captures nonlinear behaviors seen in materials, especially metals under dynamic loading conditions. This modification is expressed as follows:

(2) $$\sigma =\left(A+B\varepsilon^{n}\right)\left(1+C\ln\varepsilon^{\cdot *}\right)e^{-\lambda \left(T-T_{r}\right)}$$

where coefficients A, B, n, C, and λ correspond to specific material properties.

#### 3.1.2. Kang’s Revised JC

Kang et al. [35] suggested an enhancement to the OJC model by incorporating the strain rate parameter C as a quadratic function of the strain rate. In their model, the authors observed that experimental results indicate that the linear interpolation used in the OJC model may not be suitable for sheet metals. They suggest that quadratic interpolation could be a more appropriate choice. This modification can be expressed as follows:

(3) $$\sigma =\left(A+B\varepsilon^{n}\right)\left(1+C_{1}\ln\varepsilon^{\cdot *}+C_{2}{\ln\varepsilon^{\cdot *}}^{2}\right)\left(1-{T^{*}}^{m}\right)$$

where $A,B,n,C_{1},C_{2},$ and m are material constants.

#### 3.1.3. Lin’s Revised JC

A significant advancement to the conventional JC model was developed by Lin et al. [19]. The enhanced JC formulation preserves the initial yield condition and work hardening elements from its predecessor, while additionally accounting for the coupled effects of thermal and rate-dependent phenomena on plastic flow characteristics. This modification is expressed as follows:

(4) $$\sigma =\left(A+{B_{1}\varepsilon +B_{2}\varepsilon }^{2}\right)\left(1+C_{1}\ln\varepsilon^{\cdot *}\right)\exp\left[\left(\lambda_{1}+\lambda_{2}\ln\varepsilon^{\cdot *}\right)\left(T-T_{r}\right)\right]$$

where $A,\;B_{1},\;B_{2},\;C_{1},\lambda_{1},$ and $\lambda_{2}$ are fitting constants derived from experimental data.

#### 3.1.4. Hou’s Revised JC

The OJC model was revised by Hou et al. [36], substituting the thermal softening expression with an exponential term to better capture temperature effects. This model effectively predicts the stress–strain behavior of alloys subjected to diverse thermal environments, extending to cases where the processing temperature falls below the reference threshold. This modification can be expressed as follows:

(5) $$\sigma =\left(A+B\varepsilon^{n}\right)\left(1+C\ln\varepsilon^{\cdot *}\right)\left(1-\lambda \frac{e^{T/T_{m}}-e^{T_{r}/T_{m}}}{e-e^{T_{r}/T_{m}}}\right)$$

where A, B, n, C, and λ are material constants.

#### 3.1.5. Tan’s Revised JC

An improved JC version was developed by Tan et al. [37], accounting for interdependent strain and strain rate effects in the rate term formulation. Building upon observed deformation measurements, a coupled quadratic formulation accounting for parameter cross-effects is established to represent strain–rate dependencies. The revised formulation can be expressed as follows:

(6) $$\sigma =\left(A+B\varepsilon^{n}\right)\left(1+C\left({\varepsilon ,\varepsilon }^{\cdot }\right)\ln\varepsilon^{\cdot *}\right)\left(1-{T^{*}}^{m}\right)$$

where A,B, n and m are material constants. The parameter $C\left({\varepsilon ,\varepsilon }^{\cdot }\right)$ is determined as:

(7) $$C\left({\varepsilon ,\varepsilon }^{\cdot }\right)=C_{0}+C_{1}\varepsilon +C_{2}\varepsilon^{2}+C_{3}\ln\varepsilon^{\cdot *}+C_{4}{\ln\varepsilon^{\cdot *}}^{2}+C_{5}\varepsilon \ln\varepsilon^{\cdot *}$$

where $C_{0},\;C_{1},\;C_{2},\;C_{3},\;C_{4},$ and $C_{5}$ are five constants.

#### 3.1.6. Shokry’s-1 Revised JC

The original JC model was advanced by Shokry [32] through the integration of coupled strain/strain–rate and strain/thermal interactions. Based on experimental observations, a linear regression framework is developed to capture the coupled influence of strain with both deformation rate and thermal conditions. This modification is represented as follows:

(8) $$\sigma =\left(A+{B_{1}\varepsilon +B_{2}\varepsilon }^{2}+{\;B_{3}\varepsilon }^{3}\right)\left(1+\left(C_{1+}C_{2}\varepsilon \right)\ln\varepsilon^{\cdot *}\right)\left(1-{T^{*}}^{\left(m_{1+}m_{2}\varepsilon \right)}\right)$$

where constants $A,\;B_{1},\;B_{2}$, $B_{3}$, $C_{1}$, $C_{2}$, $m_{1},$ and $m_{2}$ are material constants.

#### 3.1.7. Tao’s Revised JC

Tao et al. [38] enhanced the conventional JC formulation through redefinition of the rate sensitivity term as a quadratic polynomial and the thermal softening component as a fourth-order polynomial function. This modification can be expressed as follows:

(9) $$\sigma =\left(A+B\varepsilon^{n}\right)\left(1+C_{1}\ln\varepsilon^{\cdot *}+C_{2}{\ln\varepsilon^{\cdot *}}^{2}\right)\left(1-T^{*a+bT^{*}+c{T^{*}}^{2}+d{T^{*}}^{3}+e{T^{*}}^{4}}\right)$$

where $A,B,\;n,\;C_{1},C_{2},a,b,c,d,$ and e are material constants.

#### 3.1.8. He’s Revised JC

He et al. [39] improved the JC formulation by integrating coupled strain–rate interactions into the constitutive framework, in which the strain rate parameter is introduced as a quadratic polynomial. This modification is represented as follows:

(10) $$\sigma =A_{1}\varepsilon^{n_{1}}.\left(1+\left(b_{1}+b_{2}\varepsilon +b_{3}\varepsilon^{2}\right)\ln\left(\varepsilon^{\cdot *}\right)\right)exp\left[\left(\lambda_{1}+\lambda_{2}\varepsilon \right)T^{*}\right]$$

where $A_{1},\;n_{1},\;b_{1},b_{2},b_{3},\lambda_{1},$ and $\lambda_{2}$ are material constants.

#### 3.1.9. Zhang’s Revised JC

Zhang et al. [40] introduced a modification to the OJC by modeling the temperature parameter as an exponential function incorporating a quadratic term in temperature based on experimental data analysis. This modification is expressed as follows:

(11) $$\sigma =\left(A+B\varepsilon^{n}\right)\left(1+C\ln\varepsilon^{\cdot *}\right)\left(1-{T^{*}}^{m\left(T\right)}\;\right)$$

where A,B, n, and C are material constants; $m\left(T\right)$ is determined as $e^{m_{0}+m_{1}T+m_{2}T^{2}}$; and $m_{0},m_{1},$ and $m_{2}$ are constants.

#### 3.1.10. Niu’s Revised JC

An improved constitutive model was developed by Niu et al. [41], integrating coupled strain–rate–temperature interactions. Analysis of experimental data reveals two key formulations: (1) a bivariate quadratic model capturing strain–rate interactions and (2) a cubic framework for nonlinear thermal–strain dependencies. Both incorporate cross-terms to quantify coupled effects on flow stress. The proposed modification can be introduced as follows:

(12) $$\sigma =\left(A\varepsilon^{B+C\varepsilon +D/\varepsilon }\right)\left(1+E\ln\varepsilon^{\cdot *}\right)\exp\left[F\left(T-T_{r}\right)\right]$$

where A, B, C, and D are material constants, and parameters E and F are given by:

(13) $$E=E_{0}+E_{1}\varepsilon +E_{2}\varepsilon^{2}+E_{3}\varepsilon^{\cdot }+E_{4}{\varepsilon^{\cdot }}^{2}+E_{5}\varepsilon \varepsilon^{\cdot }$$

(14) $$F=F_{0}+F_{1}\varepsilon +F_{2}\varepsilon^{\cdot }+F_{3}\left(T-T_{r}\right)+F_{4}\varepsilon^{3}+F_{5}{\varepsilon^{\cdot }}^{3}+F_{6}{\left(T-T_{r}\right)}^{3}+F_{7}{\varepsilon \varepsilon^{\cdot }\left(T-T_{r}\right)}^{3}$$

where $E_{i}$ and $F_{j}$ for i = 0 to 5 and j = 0 to 7 are constants.

#### 3.1.11. Chakrabarty’s Revised JC

Chakrabarty et al. [42] proposed a modification for the OJC model by considering more constants with the strain rate in which the effect of viscous drag is considered. The proposed modification can be given as follows:

(15) $$\sigma =\left(A+B\varepsilon^{n}\right)\left[1+C\ln\frac{\varepsilon^{\cdot }}{\varepsilon_{0}^{.}}{\left(\frac{\varepsilon^{\cdot }}{\varepsilon_{c}^{.}}\right)}^{D}\right]\left(1-{T^{*}}^{m}\;\right)$$

where constants $A,\;B,n,\;C,\varepsilon_{c}^{.},D,$ and m are material constants.

#### 3.1.12. Qian’s Revised JC

The OJC framework was improved by Qian et al. [43] through integration of strain-dependent rate sensitivity and thermal coupling effects. Experimental analysis supports a quadratic formulation incorporating strain–strain rate interdependencies, complemented by a linear framework for strain–temperature coupling effects. The proposed modification can be written as follows:

(16) $$\sigma =\left(A+B\varepsilon^{n}\right)\left(1+C\left(\varepsilon ,\ln\varepsilon^{\cdot *}\right)\ln\left(\varepsilon^{\cdot *}\right)\right)\left(1-m_{0}{T^{*}}^{m_{1}+m_{2}\varepsilon }\;\right)$$

where $A,\;B,\;n,m_{0},m_{1},$ and $m_{2}$ are material constants, and parameter C is given by:

(17) $$C\left(\varepsilon ,\ln\left(\varepsilon^{\cdot *}\right)\right)=C_{0}+C_{1}\varepsilon +C_{2}\varepsilon^{2}+C_{3}\varepsilon \ln\varepsilon^{\cdot *}+C_{4}\ln\varepsilon^{\cdot *}+C_{5}\ln{\varepsilon^{\cdot *}}^{2}$$

where $C_{0},\;C_{1},\;C_{2}$, $C_{3},\;C_{4},$ and $C_{5}$ are constants.

#### 3.1.13. Liu’s Revised JC

Liu et al. [44] modified the OJC formulation through incorporation of strain–rate coupling effects, in which a quadratic regression model is proposed to consider the interaction between strain and strain rate. The proposed modification can be given as follows:

(18) $$\sigma =\left(\frac{A\times \varepsilon }{B+\varepsilon }\right)\left(1+C\left(\varepsilon ,\ln\varepsilon^{\cdot *}\right)\ln\left(\varepsilon^{\cdot *}\right)\right)\left(1-T^{*m\;}\;\right)$$

where A, B, and m are material constants. Parameter C is determined as follows:

(19) $$C\left(\varepsilon ,\ln\left(\varepsilon^{\cdot *}\right)\right)=\exp\left(C_{0}+C_{1}\varepsilon +C_{2}\varepsilon^{2}+C_{3}\varepsilon \ln\varepsilon^{\cdot *}+C_{4}\ln\varepsilon^{\cdot *}+C_{5}\ln{\varepsilon^{\cdot *}}^{2}\right)$$

and $C_{0},\;C_{1},\;C_{2}$, $C_{3},\;C_{4},$ and $C_{5}$ are constants.

#### 3.1.14. Yu’s Revised JC

An improved JC variant was developed by Yu et al. [45], integrating rate–temperature coupling mechanisms and employing a quadratic formulation to quantify interdependencies between plastic deformation and thermal conditions. The proposed modification can be written as follows:

(20) $$\sigma =\left(A+{B_{1}\varepsilon +B_{2}\varepsilon }^{2}\right)\left(1+C_{1}\ln\varepsilon^{\cdot *}\right)\exp\left[\left(\lambda_{1}+\lambda_{2}\ln\varepsilon^{\cdot *}+\lambda_{3}\ln{\varepsilon^{\cdot *}}^{2}\right)\left(T-T_{r}\right)\right]$$

where $A,\;B_{1},\;B_{2},\;C_{1},\lambda_{1},\lambda_{2},$ and $\lambda_{3}$ are material constants.

#### 3.1.15. Shokry’s-2 Revised JC

A generalized JC version was established by Shokry et al. [48], accounting for coupled thermomechanical deformation effects. The modified model utilizes polynomial regression to characterize strain–rate dependencies and thermal–strain interactions, providing enhanced fidelity over the JC model’s linear simplifications. The proposed generic modification can be given as follows:

(21) $$\sigma =\left(\sum_{i=0}^{3}A_{i}\varepsilon^{i}\right)\left(1+\left(\sum_{i=0}^{2}\sum_{j=0}^{2}C_{ij\;}\varepsilon^{i}\varepsilon^{\cdot j}\right)\ln\varepsilon^{\cdot *}\right)\exp\left[\left(\sum_{i=0}^{2}\sum_{j=0}^{2}\sum_{k=0}^{2}{m_{ijk}\;\varepsilon }^{i}\varepsilon^{\cdot j}T^{*k}\right)T^{*}\right]$$

where $A_{i}$, $C_{ij\;}$, and $m_{ijk}$ give four, nine, and twenty-seven constants, respectively.

### 3.2. Fields–Backofen (FB) Model

In 1957, Fields and Backofen (FB) introduced their well-known phenomenological model for predicting flow behavior during hot deformation [49]. This model, commonly referred to as the FB model, is expressed as follows:

(22) $$\sigma =K\varepsilon^{n}\;{\varepsilon^{\cdot }}^{m}$$

The parameters K, n, and m are material constants that define key aspects of a material’s deformation behavior. K is the strength coefficient, indicating the stress level at unit strain without hardening. n is the work hardening exponent, describing how the material strengthens with plastic deformation. m is the strain rate sensitivity exponent, reflecting how flow stress varies with the rate of deformation. These constants are primarily defined as described in [75].

(23) $$K=K_{0}+K_{1}\ln\varepsilon^{\cdot }+K_{2}/T$$

(24) $$n=n_{0}+n_{1}\ln\varepsilon^{\cdot }+\frac{n_{2}}{T}$$

(25) $$m=m_{0}+m_{1}/T$$

where $K_{0},\;K_{1},\;K_{2},\;n_{0},\;n_{1},\;n_{2},\;m_{0},$ and $m_{1}$ are material constants.

The Fields–Backofen formulation serves as a fundamental framework for characterizing thermomechanical stress response in high-temperature forming operations. While valuable, this approach fails to account for critical microstructural evolution mechanisms, including dynamic crystallization, coarsening effects, and solid-state phase changes. The model’s foundation in a basic exponential stress–rate correlation often proves inadequate for representing the sophisticated deformation kinetics exhibited by materials under thermal processing conditions. Additionally, it often requires material-specific parameters that may not generalize well across different alloys or deformation conditions, reducing its predictive capability for diverse processing scenarios. Therefore, several modifications were introduced.

#### 3.2.1. Zhang’s Revised FB

Zhang [54] introduced a modification to the FB model by incorporating a softening term. This modification is represented as follows:

(26) $$\sigma =K\varepsilon^{n}\;{\varepsilon^{\cdot }}^{m}\exp\left(bT+S\varepsilon \right)$$

where b and S are two constants, and K, n, and m are given as in the FB model.

#### 3.2.2. Li’s Revised FB

A modified FB formulation was developed by Li et al. [55], accounting for strain–rate and temperature coupling mechanisms. Through quadratic approximation, the modified approach characterizes the synergistic effects of loading rate and thermal conditions. This modification can be expressed as follows:

(27) $$\sigma =K\varepsilon^{n}\;{\varepsilon^{\cdot }}^{m}$$

where K, n, and m are given as follows:

(28) $$K=K_{0}+K_{1}T+K_{2}\ln\varepsilon^{\cdot }+K_{3\;}T^{2}+K_{4\;}{\ln\varepsilon^{\cdot }}^{2}+K_{5}T\ln\varepsilon^{\cdot }$$

(29) $$n=n_{0}+n_{1}T+n_{2}\ln\varepsilon^{\cdot }+n_{3\;}T^{2}+n_{4\;}{\ln\varepsilon^{\cdot }}^{2}+n_{5}T\ln\varepsilon^{\cdot }$$

(30) $$m=m_{0}+m_{1}T+m_{2}\ln\varepsilon^{\cdot }+m_{3\;}T^{2}+m_{4\;}{\ln\varepsilon^{\cdot }}^{2}+m_{5}T\ln\varepsilon^{\cdot }$$

where $K_{i}$, $n_{i}$, and $m_{i}$ with i=0 to 5 are constants.

#### 3.2.3. Guoliang’s Revised FB

Guoliang et al. [56] suggested an enhancement to the FB model by incorporating the coupling effect between strain rate and temperature. This revised framework adopts a quadratic function to quantify rate–temperature interdependencies. This modification can be expressed as follows:

(31) $$\sigma =K\varepsilon^{n}\;{\varepsilon^{\cdot }}^{m}\exp\left(bT+S\varepsilon \right)$$

where K, n, and m are given as follows:

(32) $$n=n_{0}+n_{1}\ln\varepsilon^{\cdot }+\frac{n_{2\;}}{T}$$

(33) $$m=m_{0}+m_{1}T$$

(34) $$S=S_{0}+S_{1}\ln\varepsilon^{\cdot }+S_{2\;}/T+S_{3\;}\ln\varepsilon^{\cdot }/T+S_{4\;}{\ln\varepsilon^{\cdot }}^{3}+S_{5}\ln\varepsilon^{\cdot }/T^{2}$$

(35) $$K=K_{0}+K_{1}\ln\varepsilon^{\cdot }+K_{2\;}/T+K_{3\;}\ln\varepsilon^{\cdot }/T+K_{4\;}{\ln\varepsilon^{\cdot }}^{3}+K_{5}\ln\varepsilon^{\cdot }/T^{2}$$

where $n_{0},\;n_{1},\;n_{2},\;m_{0},\;m_{1}$, $K_{i},$ and $S_{i}$ are constants with i=0 to 5.

#### 3.2.4. Huang’s Revised FB

The FB model was improved by Huang et al. [76] by accounting for coupled temperature and deformation-rate effects. Three regression schemes capture deformation rate and thermal linkages: m follows cubic dependence, K adheres to quadratic trends, while n exhibits linear correlation. The modification they proposed is expressed as follows:

(36) $$\sigma =K\varepsilon^{n}\;{\varepsilon^{\cdot }}^{m}$$

where K, n, and m are given by the following expressions:

(37) $$K=K_{0}+K_{1}\ln\varepsilon^{\cdot }+K_{2}/T+K_{3\;}{\ln\varepsilon^{\cdot }}^{2}$$

(38) $$n=n_{0}+n_{1}\ln\varepsilon^{\cdot }+n_{2}/T$$

(39) $$m=m_{0}+m_{1}T+m_{2\;}T^{2}+m_{3}T^{3}$$

where $K_{0},\;K_{1},\;K_{2},\;{K_{3},\;n}_{0},\;n_{1},\;n_{2},\;m_{0},\;m_{1},\;m_{2},$ and $m_{3}$ are constants.

#### 3.2.5. Fu’s Revised FB

Fu et al. [57] enhanced the Fields–Backofen framework through incorporation of triaxial strain–rate–thermal interdependencies. Analysis reveals that S scales thermally and mechanically via a power–law relationship with temperature, plastic strain, and deformation rate. The proposed modification can be written as follows:

(40) $$\sigma =K\varepsilon^{n}\;{\varepsilon^{\cdot }}^{m}{\left[F\left(T,\varepsilon ,\varepsilon^{\cdot }\right)\right]}^{S}$$

where K, n, and m are given as follows:

(41) $$\left[F\left(T,\varepsilon ,\varepsilon^{\cdot }\right)\right]=A\exp\left(B\varepsilon \right)$$

(42) $$A=\frac{a_{1}}{T}+a_{2}$$

(43) $$B=b_{1}\log\left(\varepsilon^{\cdot }/\varepsilon^{\cdot *}\right)+b_{2}$$

(44) $$S=c_{1}+c_{2}\log\varepsilon^{\cdot }+c_{3}/T$$

(45) $$K=d_{1}+d_{2}{\ln\varepsilon^{\cdot }}^{2}+d_{3}/T$$

(46) $$n=e_{1}+e_{2}\ln\varepsilon^{\cdot }+e_{3}/T$$

(47) $$m=f_{1}+f_{2}/T$$

where $a_{1},a_{2},b_{1},b_{2},c_{1},c_{2,}c_{3},d_{1},d_{2,}d_{3},e_{1},e_{2,}e_{3},f_{1},$ and $f_{2}$ are constants.

#### 3.2.6. Shokry’s Revised FB

Shokry [58] proposed a modification to the FB model by adding a temperature term and accounting for the interaction between strain, strain rate, and temperature. The modified framework retains the OJC model’s normalized strain–rate formulation while augmenting the FB model with a dimensionless thermal softening term. Parameter n follows a strain-dependent quadratic function, whereas m is derived from a bivariate quadratic regression incorporating strain and deformation rate. For parameter D, a cubic function captures the triaxial coupling of strain, loading rate, and thermal conditions. The modified formulation can be expressed as follows:

(48) σ=Kεn(ε) ε·ε°·m(ε,ε·)TTrDε,ε·,T

(49) $$n\left(\varepsilon \right)=n_{0}+n_{1}\varepsilon +n_{2}\varepsilon^{2}$$

(50) $$m\left(\varepsilon ,\varepsilon^{\cdot }\right)=m_{0}+m_{1}\varepsilon +m_{2}\ln\varepsilon^{\cdot }+m_{3}\;\varepsilon \ln\varepsilon^{\cdot }+m_{4}\varepsilon^{2}+m_{5}{\ln\varepsilon^{\cdot }}^{2}+m_{6}{\;\varepsilon }^{2}{\ln\varepsilon^{\cdot }}^{2}$$

(51) $$D\left(\varepsilon ,\varepsilon^{\cdot *},T\right)=D_{0}+D_{1}\varepsilon +D_{2}\ln\varepsilon^{\cdot }+D_{3}T+D_{4}\;\varepsilon \;\ln\varepsilon^{\cdot }+D_{5}\;\varepsilon^{2}+D_{6}{\ln\varepsilon^{\cdot }}^{2}+D_{7}{\;\varepsilon }^{2}{\ln\varepsilon^{\cdot }}^{2}$$

where $n_{i}$, $m_{j}$, $D_{K}$, i=0 to 2, j=0 to 6, and k=0 to 7 are constants.

### 3.3. Khan–Huang–Liang (KHL) Model

In 1999, Khan, Huang, and Liang [59] introduced their well-known phenomenological model to predict flow behavior under varying strain rates and temperatures. The proposed model can be introduced as follows:

(52) $$\sigma =\left[A+B{\left(1-\frac{\ln\varepsilon^{\cdot }}{\ln D_{0}^{p}}\right)}^{n_{1}}\varepsilon^{n_{0}}\right]e^{C\ln\varepsilon^{\cdot }\;}\left(1-{T^{*}}^{m}\right)$$

In the KHL model, the material constants A, B, $n_{1}$, $n_{0}$, C, and m, along with an arbitrarily chosen reference strain rate $D_{0}^{p}$, are used to characterize the hot flow behavior of materials under different temperatures and strain rates. Specifically, A introduces yield stress, and B and $n_{0}$ represent the hardening coefficient and exponent, capturing the strain hardening behavior. The parameters C and $n_{1}$ account for strain rate sensitivity, while m reflects the thermal softening effect. These constants collectively enable the model to accurately describe the material’s response during high-temperature deformation.

The KHL model, while prevalent in hot deformation studies, employs oversimplified relationships that cannot fully describe the coupled effects of fundamental mechanisms (e.g., crystalline slip, thermally activated diffusion, and grain boundary accommodation) occurring during thermal processing. Such limitations have motivated several methodological improvements.

#### 3.3.1. Sim’s Revised KHL

An advancement to the KHL formulation was proposed in [62], accounting for interdependent strain–rate and temperature relationships. This advanced framework utilizes quadratic and cubic approximations to quantify interdependencies between plastic strain and loading rate. The proposed modification can be written as follows:

(53) $$\sigma =\left[A+B{\left(1-\frac{\ln\varepsilon^{\cdot }}{\ln D_{0}^{p}}\right)}^{n_{1}}\varepsilon^{n_{0}}\right]e^{C\ln\varepsilon^{\cdot }\;}\left(1-{T^{*}}^{m\left(\varepsilon ,\varepsilon^{\cdot }\right)}\right)$$

where

(54) $$m\left(\varepsilon ,\varepsilon^{\cdot }\right)=D_{0}\left(\varepsilon^{\cdot }\right)+D_{1}\left(\varepsilon^{\cdot }\right)\varepsilon +D_{2}\left(\varepsilon^{\cdot }\right)\varepsilon^{2}$$

(55) $$D_{0}\left(\varepsilon^{\cdot }\right)=\alpha_{0}+\alpha_{1}\ln\varepsilon^{\cdot *}+\alpha_{2}{\ln\varepsilon^{\cdot *}}^{2}+\alpha_{3}{\ln\varepsilon^{\cdot *}}^{3}$$

(56) $$D_{1}\left(\varepsilon^{\cdot }\right)=\beta_{0}+\beta_{1}\ln\varepsilon^{\cdot *}+\beta_{2}{\ln\varepsilon^{\cdot *}}^{2}$$

(57) $$D_{2}\left(\varepsilon^{\cdot }\right)=\gamma_{0}+\gamma_{1}\ln\varepsilon^{\cdot *}+\gamma_{2}{\ln\varepsilon^{\cdot *}}^{2}$$

and $\alpha_{0},\;\alpha_{1},\;\alpha_{2},\;\alpha_{3}$, $\beta_{0}$, $\beta_{1}$, $\beta_{2}$, $\gamma_{0}$, $\gamma_{1}$, and $\gamma_{2}$ are constants.

#### 3.3.2. Chen’s Revised KHL

Chen et al. [63] enhanced the KHL framework by integrating thermal–strain coupling mechanisms. The proposed modification can be expressed as follows:

(58) $$\sigma =\left[A+B{\left(1-\frac{\ln\varepsilon^{\cdot }}{\ln D_{0}^{p}}\right)}^{n_{1}}{\left(\frac{T_{0}-T/2}{T_{0}}\right)}^{n_{2}}\varepsilon^{n_{0}}\right]e^{C\ln\varepsilon^{\cdot }\;}\left(1-{T^{*}}^{m}\right)$$

where A, B, $n_{1}$, $n_{0}$, C, m, and $D_{0}^{p}$ are defined as in the KHL model, and $n_{2}$ is a constant.

#### 3.3.3. Singh’s Revised KHL

Singh et al. [64] introduced a modification to the KHL model by incorporating the coupling effect between temperature and strain by incorporating a temperature term into the hardening part. This proposed modification is expressed as follows:

(59) $$\sigma =\left[A+B\exp\left(C_{0}\ln\varepsilon^{\cdot *}\right)\;{T^{*}}^{m_{0}}\varepsilon^{n_{0}}\right]e^{C_{1}\ln\varepsilon^{\cdot *}\;}\left(1-{T^{*}}^{m_{1}}\right)$$

where A, B, and $n_{0}$ are defined as in the KHL model, and $C_{0}$, $C_{1}$, $m_{0}$, and $m_{1}$ are constants.

### 3.4. Zerilli–Armstrong (ZA) Model

Zerilli–Armstrong [65] introduced their well-known physical model in 1987, presenting two proposed models for FCC and BCC metal crystal structures. These models can be expressed as follows:

(60) $$\sigma =C_{0}+C_{1}\varepsilon^{0.5}\exp\left(-C_{2}T+C_{3}T\ln\varepsilon^{\cdot }\right)\;\;\;\;\mathrm{for}\;\mathrm{FCC}$$

(61) $$\sigma =C_{0}+C_{1}\exp\left(-C_{2}T+C_{3}T\ln\varepsilon^{\cdot }\right)+C_{4}\varepsilon^{n}\;\;\;\mathrm{for}\;\mathrm{BCC}$$

The parameters $C_{0}$, $C_{1}$, $C_{2}$, $C_{3}$, $C_{4},$ and n are material constants in the model. Specifically, $C_{0}$ represents the initial yield stress, while $C_{1}$ serves as a scaling factor for the thermally activated stress component. $C_{2}$ defines the thermal softening coefficient, and $C_{3}$ accounts for the strain rate sensitivity. Additionally, $C_{4}$ is the strain hardening coefficient, and n denotes the strain hardening exponent. These constants together describe how the material responds under different thermal and mechanical loading conditions.

Modifications to the Zerilli–Armstrong (ZA) model are needed to better predict hot deformation behavior because the original model simplifies the relationship between temperature, strain rate, and material properties. It does not fully account for complex factors like grain size, phase transformations, strain hardening or softening, and material-specific characteristics. These factors can significantly influence deformation, making it necessary to adjust the model to improve its accuracy and applicability across different materials and conditions.

#### 3.4.1. Samantaray’s Revised ZA

Samantaray et al. [77] introduced a well-known modification to the ZA model by incorporating the coupling effects between strain and temperature, as well as between temperature and strain rate, in which both are modeled using linear regression. This modification can be expressed as follows:

(62) $$\sigma =\left(C_{1}+C_{2}\varepsilon^{n}\right)\exp\left[-\left(C_{3}+C_{4}\varepsilon \right)T^{*}+\left(C_{5}+C_{6}T^{*}\right)\ln\varepsilon^{\cdot *}\right]$$

where $C_{1}$, $C_{2}$, $n,\;C_{3}$, $C_{4}$, $C_{5},$ and $C_{6}$ are material constants, and $T^{*}=T-T_{r}$.

#### 3.4.2. Sim’s Revised ZA

An improved ZA version was developed in [62], accounting for coupled strain (quadratic)–temperature interactions. In this proposed modified model, strain is modeled using quadratic regression to represent the interaction with temperature. This modification can be formulated as:

(63) $$\sigma =\left(A_{1}+A_{2}\varepsilon {+A}_{3}\varepsilon^{2}\right)\exp\left[-\left(B_{1}+B_{2}\varepsilon {+B}_{3}\varepsilon^{2}\right)T^{*}+\left(C_{1}+C_{2}T^{*}\right)\ln\varepsilon^{\cdot *}\right]$$

where $A_{1}$, $A_{2}$, $A_{3}$, $B_{1}$, $B_{2}$, $B_{3}$, $C_{1}$, and $C_{2}$ are material constants.

#### 3.4.3. Li’s Revised ZA

An improved ZA version was developed in [78], capturing both mechanical rate sensitivity and thermally coupled strain behavior. A fifth-degree polynomial strain function was utilized to characterize the combined deformation–rate–thermal relationships. The proposed modification can be expressed as follows:

(64) $$\sigma =\exp\left[I_{1}+S_{1}T^{*}+\left(C_{5}+C_{6}T^{*}\right)\ln\varepsilon^{\cdot *}\right]$$

with

(65) $$\left[\begin{array}{c} I_{1}=i_{0}+i_{1}\varepsilon {+i}_{2}\varepsilon^{2}{+i}_{3}\varepsilon^{3}{+i}_{4}\varepsilon^{4}{+i}_{5}\varepsilon^{5}\\ S_{1}=s_{0}+s_{1}\varepsilon {+s}_{2}\varepsilon^{2}{+s}_{3}\varepsilon^{3}{+s}_{4}\varepsilon^{4}{+s}_{5}\varepsilon^{5}\\ C_{5}=e_{0}+e_{1}\varepsilon {+e}_{2}\varepsilon^{2}{+e}_{3}\varepsilon^{3}{+e}_{4}\varepsilon^{4}{+e}_{5}\varepsilon^{5}\\ C_{6}=f_{0}+f_{1}\varepsilon {+f}_{2}\varepsilon^{2}{+f}_{3}\varepsilon^{3}{+f}_{4}\varepsilon^{4}{+f}_{5}\varepsilon^{5} \end{array}\right]$$

where $i_{i}$, $s_{i}$, $e_{i}$, and $f_{i}$ with i=0 to 5 are constants.

#### 3.4.4. Huang’s Revised ZA

An enhanced ZA framework was developed in [76], integrating both heat–strain interactions and thermal influences on deformation kinetics. In the proposed modified model, strain is represented using a cubic regression to capture its interaction with temperature, while temperature is also modeled with a cubic regression to reflect its interaction with strain rate. This modification can be expressed as follows:

(66) $$\sigma =\left(A_{0}+A_{1}\varepsilon {+A}_{2}\varepsilon^{2}{+A}_{3}\varepsilon^{3}\right)\exp\left[-\left(B_{0}+B_{1}\varepsilon {+B}_{2}\varepsilon^{2}{+B}_{3}\varepsilon^{3}\right)T^{*}+\left(C_{0}+C_{1}T^{*}+C_{2}T^{*2}+C_{3}T^{*3}\right)\ln\varepsilon^{\cdot *}\right]$$

where $A_{i}$, $B_{i}$, and $C_{i}$ with i=0 to 3 are constants.

#### 3.4.5. Shokry’s Revised ZA

Shokry’s work [58] extended the ZA constitutive framework by simultaneously coupling deformation mechanics, loading kinetics, and thermal response. Based on experimental data analysis, strain is modeled using a cubic regression to represent the hardening term. To capture the interaction with temperature, both temperature and strain are modeled using quadratic regression. Additionally, the interaction with strain rate is represented through a quadratic regression involving strain, strain rate, and temperature. This proposed modification can be expressed as follows:

(67) $$\sigma =A\left(\varepsilon \right)\exp\left\{B\left(\varepsilon ,T^{*}\right)T^{*}+C\left(\varepsilon ,T^{*},\varepsilon^{\cdot *}\right)\ln\varepsilon^{\cdot *}\right\}$$

(68) $$\begin{array}{l} A\left(\varepsilon \right)=A_{0}+A_{1}\varepsilon +A_{2}\varepsilon^{2}+A_{3}\varepsilon^{3}\\ B\left(\varepsilon ,T^{*}\right)=B_{0}+B_{1}\varepsilon +B_{2}T^{*}+B_{3}\varepsilon \;T^{*}+B_{4}\varepsilon^{2}+B_{5}{T^{*}}^{2}+B_{6}\varepsilon^{2}{T^{*}}^{2}\\ C\left(\varepsilon ,T^{*},\varepsilon^{\cdot *}\right)=C_{0}+C_{1}\varepsilon +C_{2}T^{*}+C_{3}\ln\varepsilon^{\cdot *}{+C}_{4}\varepsilon \ln\varepsilon^{\cdot *}+C_{5}\varepsilon^{2}+C_{6}{\ln\varepsilon^{\cdot *}}^{2}+C_{7}\varepsilon^{2}{\ln\varepsilon^{\cdot *}}^{2} \end{array}$$

where and $A_{i}$, $B_{j}$, and $C_{k}$ with i=0 to 3, j=0 to 6, and k=0 to 7 are constants.

## 4. Results

### 4.1. Determination of Model Constants

Accurate determination of material constants that constitute models for predicting flow behavior during hot deformation is crucial for several reasons. First, these constants directly influence the precision of simulations used in manufacturing processes like forging, rolling, and extrusion, where the material undergoes significant changes in shape and temperature. Reliable constants ensure that the models can predict the material’s response considering variations in: (i) thermal state, (ii) deformation kinetics, and (iii) stress state. This enables optimization of process parameters, leading to better product quality, reduced energy consumption, and minimized defects. Moreover, accurate material constants help in the development of new alloys and materials by providing insights into their deformation characteristics, thereby guiding the design of processes for improved performance and efficiency. Inaccurate values, on the other hand, can lead to suboptimal processing conditions, costly errors, and compromised material properties.

An inverse analysis approach employing MATLAB optimization is adopted to identify material parameters. This iterative scheme, initialized with user-defined values, applies the Levenberg–Marquardt algorithm [70] to reconcile numerical predictions with empirical stress data through residual error reduction, effectively capturing the complex thermomechanical response of metallic systems. Strain, strain rate, and temperature are treated as predictors, while stresses are treated as responses. The Levenberg–Marquardt algorithm is given by [79]:

(69) $$x_{k+1}=x_{k}-{\left[J^{T}J+\mu I\right]}^{-1}J^{T}\left(z-h\left(x_{k}\right)\right)$$

where x denotes a vector that includes model constants, i.e., $x={\left[x_{1},\;{\;x}_{2},\;\ldots ..,\;x_{t}\right]}^{T}$, and t represents the total number of constants. The parameter μ presents a scalar value, and I is the identity matrix. With μ=0, Levenberg–Marquardt converts to the Gauss–Newton algorithm. The parameter z presents a vector that includes experimental stresses, i.e., $z={\left[z_{1},\;{\;z}_{2},\;\ldots ..,\;z_{N}\right]}^{T}$, and N is the total number of measurements of stresses. The parameter $h\left(x_{k}\right)$ denotes a vector containing the predicted stresses at $x_{k}$. The parameter J defines a Jacobian matrix sized N×t, containing the derivatives of $h\left(x_{k}\right)$ with regard to $x_{k}$ and is introduced as follows:

(70) $$J=\left[\begin{array}{ccc} \frac{{\partial h}_{1}}{{\partial x}_{1}} & \cdots & \frac{{\partial h}_{1}}{{\partial x}_{t}}\\ \vdots & \ddots & \vdots \\ \frac{{\partial h}_{N}}{{\partial x}_{1}} & \cdots & \frac{{\partial h}_{N}}{{\partial x}_{t}} \end{array}\right]$$

### 4.2. Evaluation of the Models

Model validation under thermomechanical processing conditions employs standard statistical indicators, including R, AARE, and RMSE. Specifically, the correlation coefficient R evaluates how closely predicted stresses align with measured values along a linear trend, with ideal predictions yielding R = 1. The AARE evaluates prediction accuracy by computing mean absolute percentage deviations from experimental measurements, revealing systematic errors in the constitutive formulation. In contrast, the RMSE emphasizes larger prediction variances through quadratic error weighting, making it particularly responsive to anomalous data points. These complementary metrics collectively assess model fidelity, where superior performance corresponds to minimized AARE/RMSE scores and maximized correlation coefficients. R, AARE, and RMSE are given as in [48]:

(71) $$R=\frac{\sum_{i}^{N}\left(\sigma_{e}-\bar{\sigma_{e}}\right)\left(\sigma_{P}-\bar{\sigma_{P}}\right)}{\sqrt{\sum_{i}^{N}{\left(\sigma_{e}-\bar{\sigma_{e}}\right)}^{2}\sum_{i}^{N}{\left(\sigma_{P}-\bar{\sigma_{P}}\right)}^{2}}}$$

(72) $$AARE=\frac{1}{N}\sum_{i}^{N}\left|\frac{\sigma_{e}-\sigma_{P}}{\sigma_{e}}\right|\times 100$$

(73) $$RMSE=\frac{1}{N}\sqrt{\sum_{i}^{N}{\left(\sigma_{e}-\sigma_{P}\right)}^{2}}$$

where $\sigma_{e}$ denotes the experimentally measured stress values with $\bar{\sigma_{e}}$ being their arithmetic mean, $\sigma_{P}$ indicates the model-predicted stresses with $\bar{\sigma_{P}}$ representing their average, and N corresponds to the sample size

#### 4.2.1. U720LI Ni-Based Alloy

**Figure 1:**
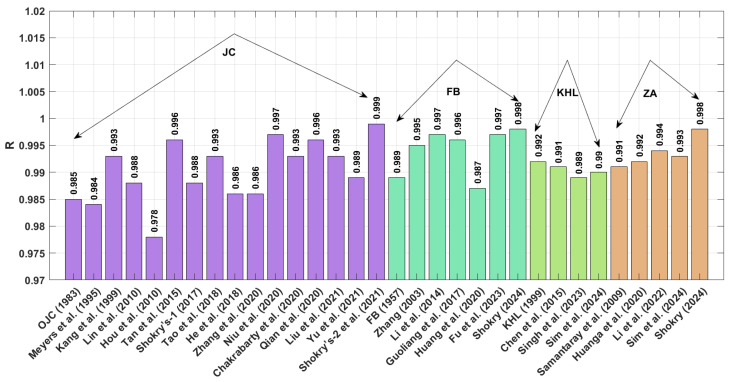
R for JC, FB, KHL, and ZA and their modified based models for U720LI alloy.

The results obtained for R (as shown in Figure 1 and presented by Equation (71) indicate that the modified–based JC proposed by Shokry et al. [48] (see Section 3.1.15) achieves the highest R value of 0.999, whereas the modified-based JC introduced by Hou et al. [36] (see Section 3.1.4) yields a highest R value of 0.978.

**Figure 2:**
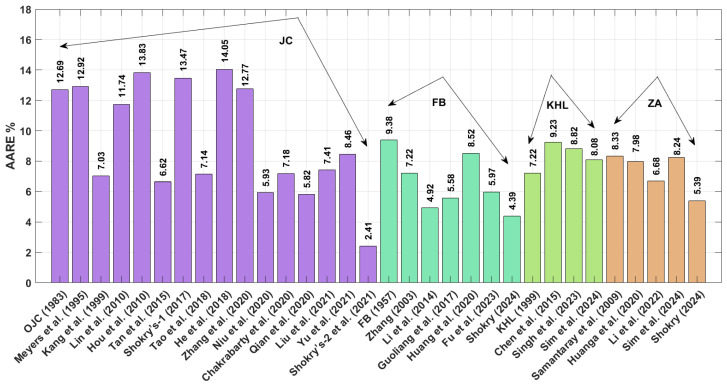
AARE% for JC, FB, KHL, and ZA and their modified models for U720LI alloy.

A comparison of the AARE values obtained using Equation (72) for the JC, FB, KHL, ZA, and their modified bases is shown in Figure 2. This figure demonstrates that the modified–based JC proposed by Shokry et al. [48] (see Section 3.1.15) yields the lowest AARE, at 2.41%, while the modified–based JC introduced by He et al. [39] (see Section 3.1.8) results in the highest AARE, at 14.05%.

**Figure 3:**
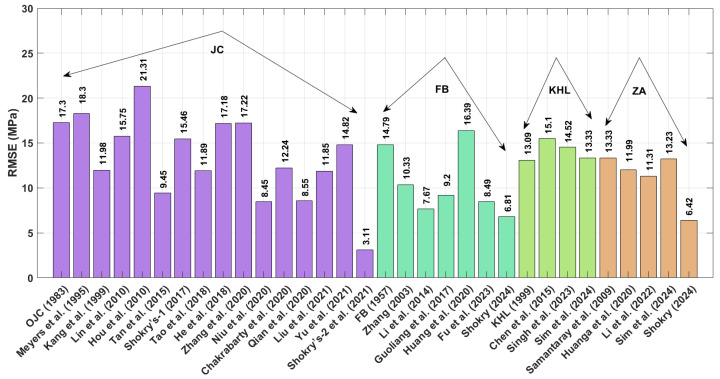
RMSE for JC, FB, KHL, and ZA and their modified models for U720LI alloy.

The RMSE values in Figure 3 (Equation (73)) demonstrate that the JC version proposed by Shokry et al. [48] (see Section 3.1.15) outperforms other models, with a minimal RMSE of 3.11 MPa. In comparison, the JC version developed by Hou et al. [36] (Section 3.1.4) yields the largest prediction errors, with an RMSE reaching 21.31 MPa.

**Figure 4:**
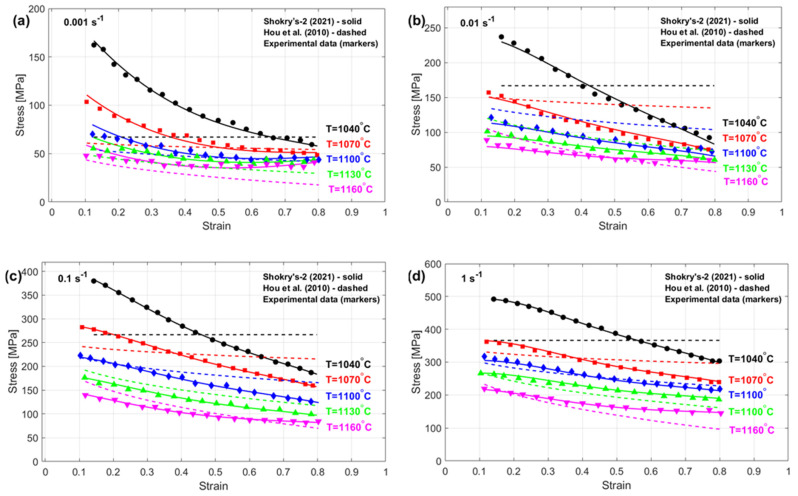
Predicted stresses by Shokry et al. [48] and Hou et al. [36] versus experimental stresses for U720LI alloy [71] at (**a**) 0.001 s^−^^1^, (**b**) 0.01 s^−^^1^, (**c**) 0.1 s^−^^1^, and (**d**) 1 s^−^^1^.

The stresses obtained using the modifications proposed by Shokry et al. [48] and Hou et al. [36], representing the best and worst results, respectively, are compared to experimental stresses considering diverse temperature–strain rate combinations, as shown in Figure 4. It is evident that the modification by Shokry et al. [48] accurately predicts the flow behavior of the U720LI alloy within the tested temperature and strain rate ranges. This can be attributed to the coupling effect between strain, strain rate, and temperature. In contrast, the modification by Hou et al. [36] fails to predict the flow behavior of the U720LI alloy, indicating its inability to capture interdependencies among thermomechanical processing variables.

**Figure 5:**
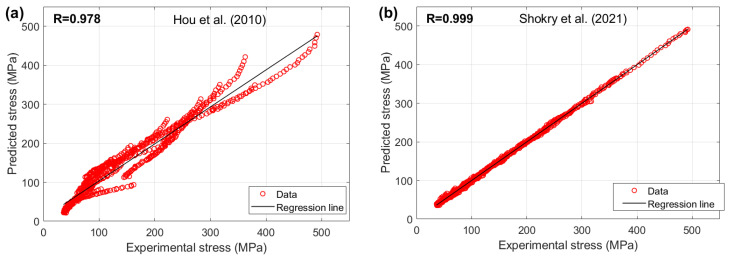
R between predicted stresses by (**a**) Hou et al. [36] and (**b**) Shokry et al. [48] versus experimental stresses for U720LI alloy.

Figure 5 compares the correlation coefficients (R) between experimental and predicted stresses for U720LI alloy using the models of Shokry et al. [48] and Hou et al. [36]. The Shokry model demonstrates exceptional agreement (R = 0.999) with experimental data, while the Hou model shows significantly lower correlation (R = 0.978).

#### 4.2.2. AA7020—Al-Based Alloy

**Figure 6:**
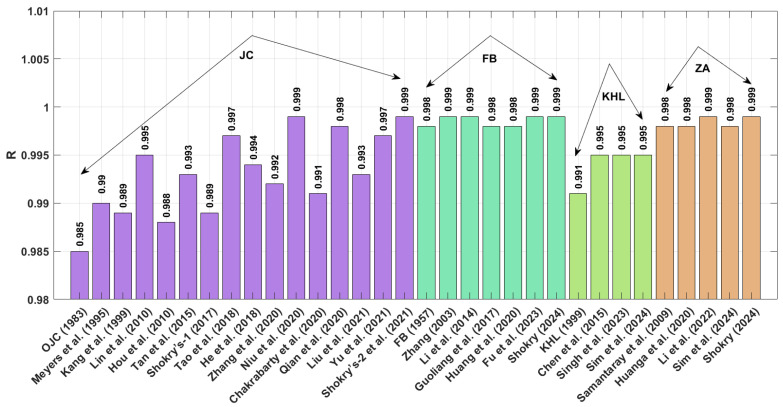
R for JC, FB, KHL, and ZA and their modified models for AA7020 alloy.

The results for R (refer to Figure 6), as expressed in Equation (71), indicate that the modified JC models presented by Shokry et al. [48] (cf. Section 3.1.15) and Niu et al. [41] (cf. Section 3.1.10), modified ZA models proposed by Shokry [58] (cf. Section 3.4.5) and Li et al. [78] (cf. Section 3.4.3), and modified FB models introduced by Zhang [54] (cf. Section 3.2.1), Li et al. [54] (cf. Section 3.2.2), and Fu et al. [57] (cf. Section 3.2.5) achieve the highest R value of 0.999. In comparison, the original JC model (refer to Section 3.1) attains the lowest R value of 0.985.

**Figure 7:**
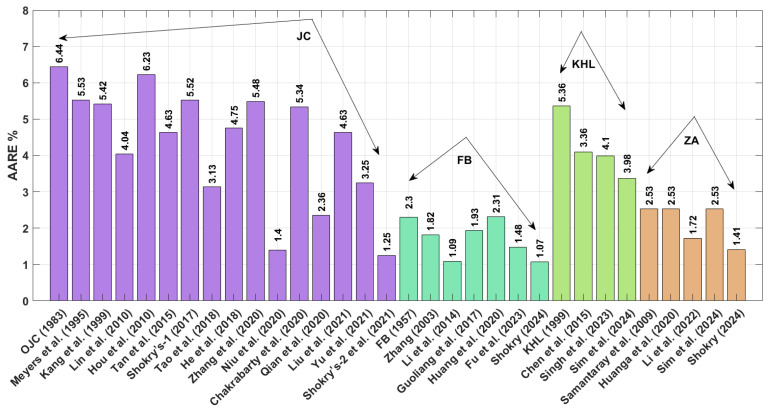
AARE% for JC, FB, KHL, and ZA and their modified models for AA7020 alloy.

The AARE performance of different material models, evaluated using Equation (72) for the JC, FB, KHL, and ZA models and their modified versions, is displayed in Figure 7. Results indicate that the FB modification by Shokry [58] (Section 3.2.6) provides the best fit to experimental data (AARE = 1.07%), contrasting sharply with the original JC model’s poor performance (AARE = 6.44%) detailed in Section 3.1.

**Figure 8:**
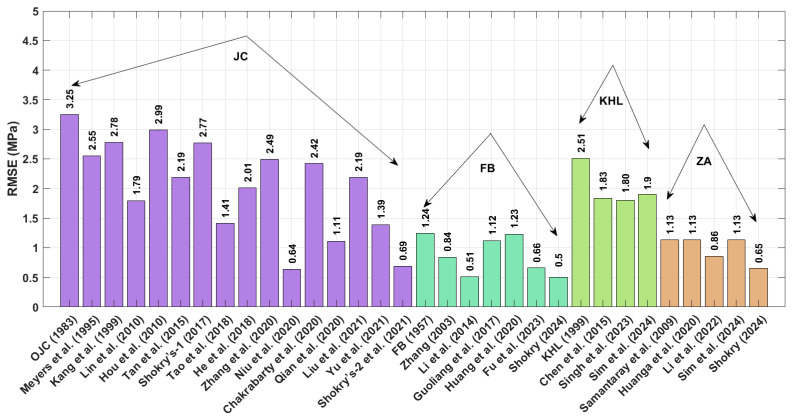
RMSE for JC, FB, KHL, and ZA and their modified models for AA7020 alloy.

The RMSE results (see Figure 8) calculated using Equation (73) indicate that the modified FB model proposed by Shokry [58] (refer to Section 3.2.6) achieved the lowest RMSE value of 0.5 MPa, while the OJC [31] model (refer to Section 3.1) yielded the highest RMSE value of 3.25 MPa.

**Figure 9:**
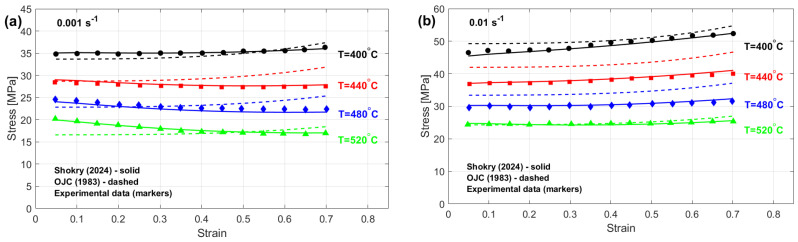

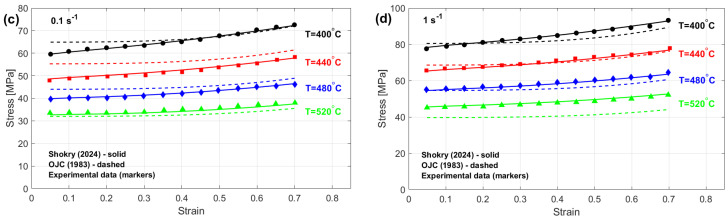
Predicted stresses by Shokry [58] and the original JC versus experimental stresses for AA7020 alloy [72] at (**a**) 0.001 s^−^^1^, (**b**) 0.01 s^−^^1^, (**c**) 0.1 s^−^^1^, and (**d**) 1 s^−^^1^.

A comparative assessment of predictive capabilities appears in Figure 9, evaluating Shokry’s high-accuracy FB adaptation [58] against the low-accuracy original JC model [31]. The modified FB approach precisely reproduces AA7020 flow stresses throughout the experimental parameter space, achieved through integrated strain–temperature–rate coupling. The OJC model’s failure to capture AA7020 deformation mechanics reflects its lack of these essential multivariable interactions.

**Figure 10:**
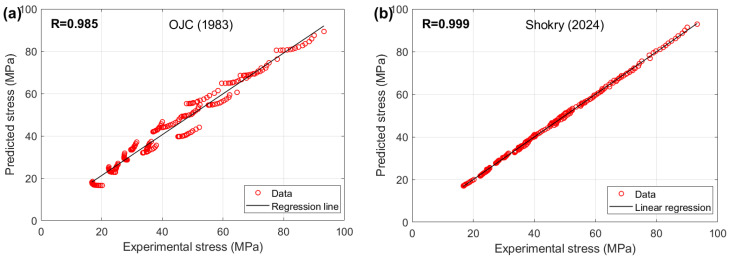
R between predicted stresses by (**a**) OJC [31] and (**b**) Shokry [58] versus experimental stresses for AA7020 alloy.

Figure 10 illustrates the correlation between the predicted stresses by the modified FB model proposed by Shokry [58] and the original JC model [31] with the experimental stresses for the AA7020 alloy. The results indicate that Shokry [58] achieved a very high correlation accuracy, with R = 0.999, whereas the original JC exhibited a lower correlation accuracy, with R = 0.985.

#### 4.2.3. Ti-4.5Al-1V-3Fe—Ti-Based Alloy

**Figure 11:**
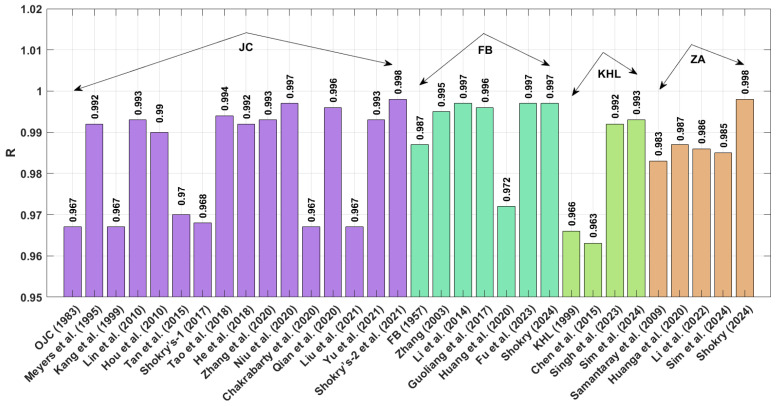
R for JC, FB, KHL, and ZA and their modified models for Ti-4.5Al-1V-3Fe alloy.

Figure 11 presents the correlation coefficients (R) for various constitutive models. Both Shokry’s enhanced ZA formulation [58] (Section 3.4.5) and the refined JC model by Shokry et al. [48] (Section 3.1.15) demonstrate exceptional predictive accuracy with R = 0.998. By contrast, Chen’s modified KHL approach [63] (Section 3.3.2) shows a significantly lower correlation (R = 0.963).

**Figure 12:**
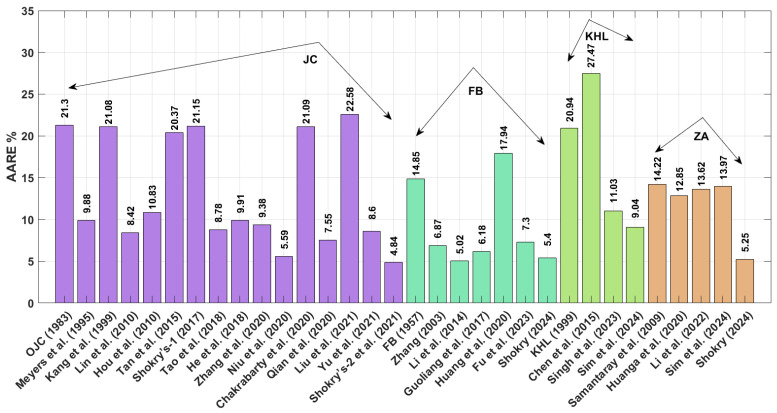
AARE% for JC, FB, KHL, and ZA and their modified models for Ti-4.5Al-1V-3Fe alloy.

The AARE performance comparison in Figure 12 reveals significant differences between models. Shokry’s refined JC framework [48] (Section 3.1.15) achieves the best agreement with experimental data (AARE = 4.84%), contrasting sharply with Chen’s KHL version [63] (Section 3.3.2), which shows the poorest fit (AARE = 27.47%).

**Figure 13:**
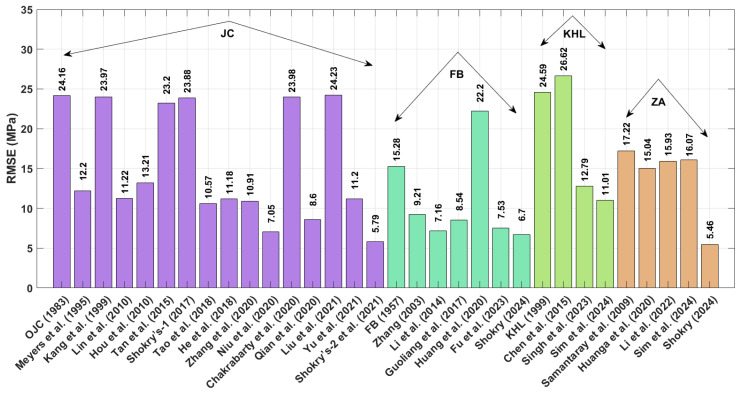
RMSE for JC, FB, KHL, and ZA and their modified models for Ti-4.5Al-1V-3Fe alloy.

The RMSE results in Figure 13, calculated using Equation (73), reveal that the modified ZA model proposed by Shokry [58] (see Section 3.4.5) achieved the lowest RMSE value of 5.46 MPa, whereas the modified KHL model, proposed by Chen et al. [63] (refer to Section 3.3.2), produced the highest RMSE value of 26.62 MPa.

**Figure 14:**
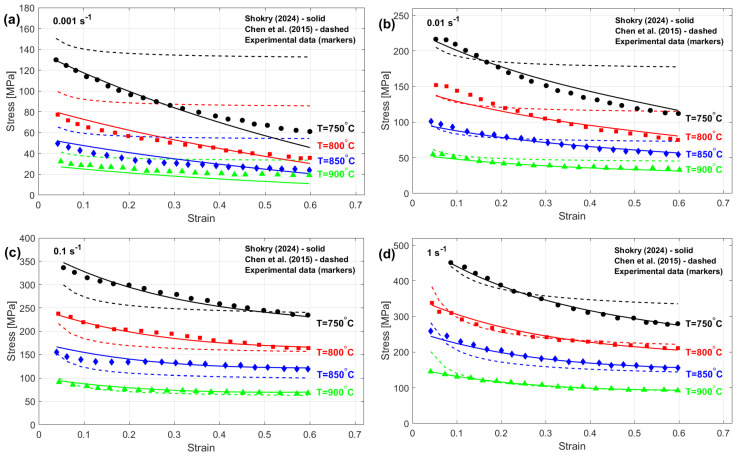
Predicted stresses by Shokry [58] and the Chen et al. [63] versus experimental stresses for Ti-4.5Al-1V-3Fe alloy [73] at (**a**) 0.001 s^−1^, (**b**) 0.01 s^−1^, (**c**) 0.1 s^−1^, and (**d**) 1 s^−1^.

The stresses predicted by the modified ZA model that is presented by Shokry [58] provide the most accurate results, and the modified KHL model, proposed by Chen et al. [63], representing the least accurate outcomes, are compared with experimental stresses considering different thermal–mechanical processing parameters, as shown in Figure 14. Shokry’s modifications [58] demonstrate an effective ability to capture the flow behavior of the Ti-4.5Al-1V-3Fe alloy within the tested temperature and strain rate ranges. Superior accuracy results from explicit accounting for interdependent strain, deformation rate, and thermal effects. In contrast, the proposed modification by Chen et al. [63] fails to accurately predict the flow behavior of the Ti-4.5Al-1V-3Fe alloy, likely due to its inability to account for these interconnected hot working parameters.

**Figure 15:**
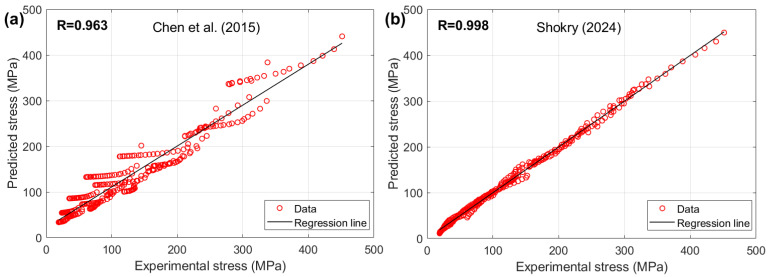
R between predicted stresses by (**a**) Chen et al. [63] and (**b**) Shokry [58] versus experimental stresses for Ti-4.5Al-1V-3Fe alloy.

Figure 15 presents the correlation between the predicted stresses by the modified ZA model proposed by Shokry [58] and the modified KHL model presented by Chen et al. [63] with the experimental stresses for the Ti-4.5Al-1V-3Fe alloy. The results indicate that Shokry [58] achieved a high correlation accuracy, with R = 0.998, whereas Chen et al. [63] exhibited a lower correlation accuracy, with R = 0.963.

#### 4.2.4. 40Cr Steel Alloy

**Figure 16:**
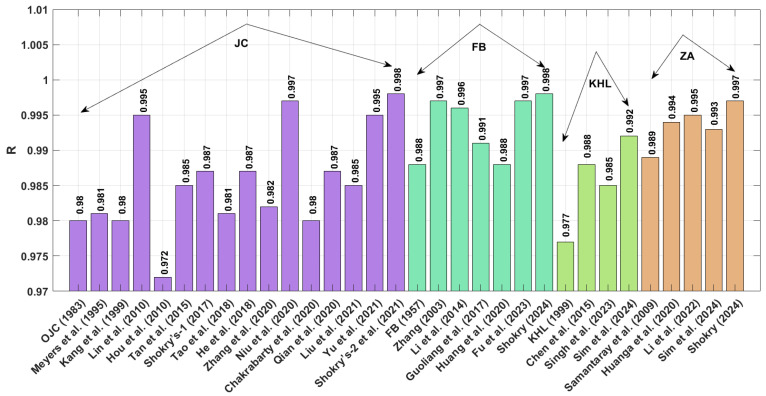
R for JC, FB, KHL, and ZA and their modified models for 40Cr steel alloy.

The R values in Figure 16 reveal that Shokry’s JC [48] and FB [58] modifications (Section 3.1.15 and Section 3.2.6, respectively) yield exceptional predictive accuracy (R = 0.998), while Hou’s JC version [36] (Section 3.1.4) produces the poorest alignment (R = 0.972) with experimental measurements.

**Figure 17:**
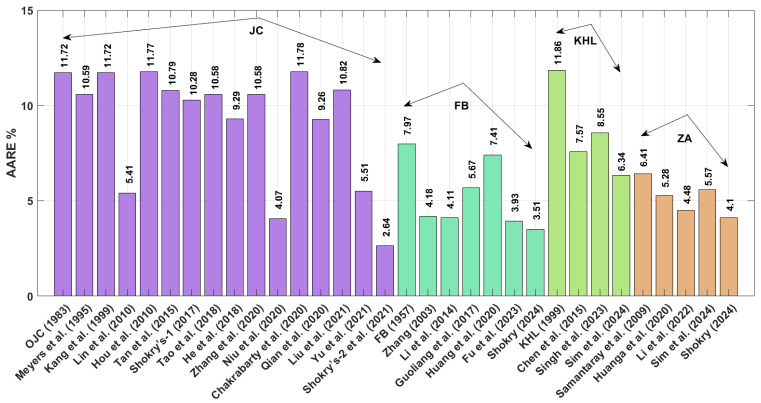
AARE% for JC, FB, KHL, and ZA and their modified models for 40Cr steel alloy.

The obtained AARE values calculated using Equation (72) for the JC, FB, KHL, and ZA models, along with their modified versions, are presented in Figure 17. This figure highlights that the improved JC version established by Shokry et al. [48] (see Section 3.1.15) achieves the lowest AARE of 2.64%. In contrast, the original KHL model (see Section 3.3) exhibits the highest AARE at 11.86%.

**Figure 18:**
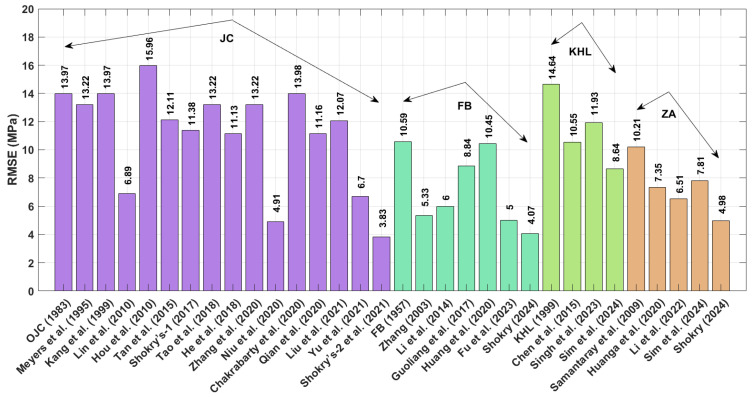
RMSE for JC, FB, KHL, and ZA and their modified models for 40Cr steel alloy.

The RMSE results in Figure 18, calculated using Equation (73), indicate that the modified ZA model proposed by Shokry et al. [48] (cf. Section 3.1.15) achieved the lowest RMSE value of 3.83 MPa, whereas the revised JC version presented by Hou et al. [36] (cf. Section 3.1.4) produced the highest RMSE value of 15.96 MPa.

**Figure 19:**
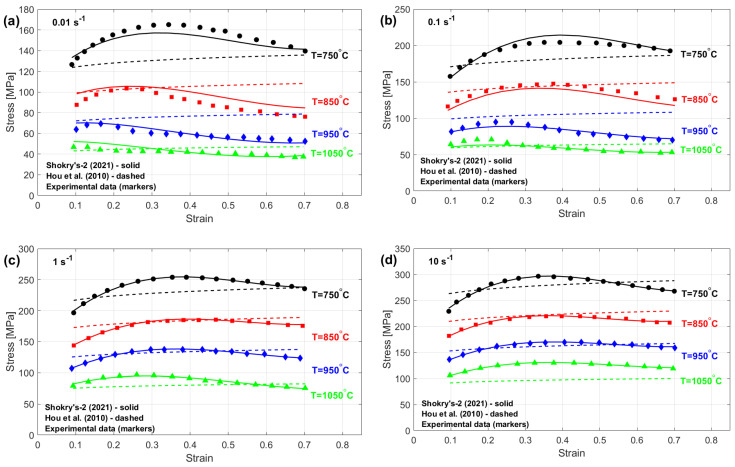
Predicted stresses by Shokry [58] and the Hou et al. [36] versus experimental stresses for 40Cr steel alloy [74] at (**a**) 0.001 s^−1^, (**b**) 0.01 s^−1^, (**c**) 0.1 s^−1^, and (**d**) 1 s^−1^.

The stresses predicted through the enhanced JC formulation proposed by Shokry et al. [48], which stands out as the most accurate, and the refined JC framework developed by Hou et al. [36], which yields the least accurate results, are compared with experimental stresses for various thermal processing and strain–rate conditions, as shown in Figure 19. Shokry’s modifications [48] effectively capture the flow behavior of the 40Cr steel alloy within the tested temperature and strain rate ranges. Such precision improvements originate from comprehensive modeling of coupled mechanical–thermal interactions. In contrast, the proposed modification by Hou et al. [36] fails to accurately predict the flow behavior of the 40Cr steel alloy, likely because they do not adequately consider these interconnected hot working parameters.

**Figure 20:**
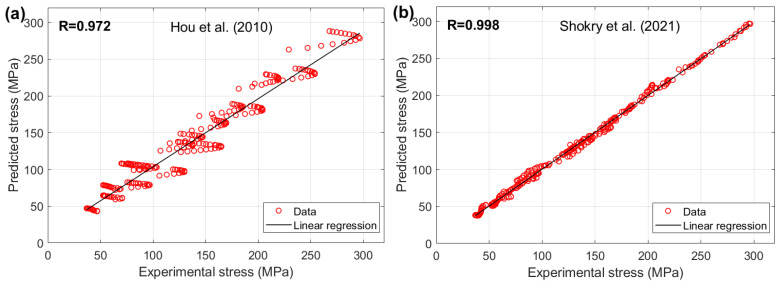
R between predicted stresses by (**a**) Hou et al. [36] and (**b**) Shokry et al. [48] versus experimental stresses for 40Cr steel alloy.

Figure 20 illustrates the correlation between the predicted stresses from the proposed JC models proposed by Shokry et al. [48] and Hou et al. [36] with the experimental stresses for the 40Cr steel alloy. The findings show that Shokry’s model [48] demonstrated a high correlation accuracy, achieving R = 0.998, while Hou et al.’s model [36] exhibited a lower correlation accuracy, with R = 0.972.

## 5. Discussions

The flow behavior of four different element-based metal alloys experiencing broad ranges of extreme heat and mechanical strain rates is investigated using phenomenological models and physical models. The four different element-based alloys are nickel-based alloy (U720LI), aluminum-based alloy (AA7020), titanium-based alloy (Ti-4.5Al-1V-3Fe), and iron-based alloy (40Cr steel). The phenomenological models are JC, FB, and KHL and their modified versions, while the physical model is ZA and its modified version.

The improved JC version developed by Shokry et al. [48] achieves the lowest AARE value of 2.41% for the U720LI alloy. Following this, the modified FB-based models by Shokry [58] and Li et al. [55] exhibit AARE values of 4.39% and 4.92%, respectively. Subsequently, the modified ZA-based model by Shokry [58] and the improved JC formulation by Niu et al. [41] yield AARE values of 5.39% and 5.93%, respectively. Considering the results obtained for RMSE for the U720LI alloy, the improved JC developed by Shokry et al. [48] achieves the lowest RMSE value of 3.11 MPa. Following this, the modified ZA-based and modified FB-based models by Shokry [58] exhibit RMSE values of 6.42 MPa and 6.81 MPa. Subsequently, the modified FB-based model by Li et al. [55] and the improved JC by Niu et al. [41] yield RMSE values of 7.67 MPa and 8.45 MPa, respectively. Regarding the results obtained for R for the U720LI alloy, the improved JC proposed by Shokry et al. [48] achieves the highest R value of 0.999. Following this, the modified ZA-based and FB-based models by Shokry [58] show an R value of 0.998. Additionally, the modified FB-based model by Li et al. [55] and the improved JC by Niu et al. [41] result in R values of 0.997 and 0.997, respectively.

The modified FB-based models proposed by Shokry [58] and Li et al. [55] achieve the lowest AARE values of 1.07% and 1.09% for the AA7020 alloy. Following this, the modified JC-based model by Shokry et al. [48] and by Niu et al. [41] and the modified ZA-based model by Shokry [58] report AARE values of 1.25%, 1.4%, and 1.41%, respectively. In terms of RMSE for the AA7020 alloy, modified FB-based models proposed by Shokry [58] and Li et al. [55] yield the lowest RMSE values of 0.5 MPa and 0.51 MPa. This is followed by the modified JC-based model by Niu et al. [41] and the modified ZA-based model proposed by Shokry [58], with RMSE values of 0.64 MPa and 0.65 MPa, respectively. Meanwhile, the modified FB-based model by Fu et al. [57] and the improved JC by Shokry et al. [48] exhibit RMSE values of 0.66 MPa and 0.69 MPa, respectively. Regarding the correlation coefficient (R) for the AA7020 alloy, the improved JC models proposed by Shokry et al. [48] and Niu et al. [41], along with the modified version of FB models by Shokry [58], Fu et al. [57], and Li et al. [55], with refined version ZA models proposed by Shokry [58] and Li et al. [78], give a common high R value of 0.999.

The improved version of JC proposed by Shokry et al. [48] achieves the lowest AARE values of 4.84% for the Ti-4.5Al-1V-3Fe alloy. Following this, the refined FB-based model and the revised ZA-based model by Shokry [58] give AARE values of 5.25% and 5.40%, respectively. Additionally, the improved JC-based model by Niu et al. [41] exhibits an AARE value of 5.59%. In terms of RMSE for the Ti-4.5Al-1V-3Fe alloy, the revised ZA models by Shokry [58] and improved JC proposed by Shokry et al. [48] yield the lowest RMSE values of 5.46 MPa and 5.79 MPa, respectively. These are followed by the revised FB-based model by Shokry [58] and the improved JC by Niu et al. [41] with RMSE values of 6.70 MPa and 7.05 MPa, respectively. Regarding the correlation coefficient (R) for the Ti-4.5Al-1V-3Fe alloy, the revised ZA models by Shokry [58] and improved JC proposed by Shokry et al. [48] yield the highest R values, with 0.998. Following this, the revised FB-based model by Shokry [58] and by Li et al. [55] and the improved JC model by Niu et al. [41] give R values of 0.997.

The improved version of JC developed by Shokry et al. [48] achieves the lowest AARE value of 2.64% for the 40Cr steel alloy. Following this, the revised FB models by Shokry [58] and Fu et al. [57] exhibit AARE values of 3.51% and 3.93%, respectively. Additionally, the improved JC model by Niu et al. [41] and the revised ZA-based models by Shokry [58] demonstrate an AARE value of 4.07% and 4.10%, respectively. For RMSE values associated with the 40Cr steel alloy, improved JC proposed by Shokry et al. [48] yields the lowest RMSE with a value of 3.80 MPa. Following this, the revised FB-based model by Shokry [58] gives an RMSE value of 4.07 MPa. These are followed by the improved JC by Niu et al. [41] and the revised ZA by Shokry [58], with RMSE values of 4.91 MPa and 4.98 MPa, respectively. Regarding the correlation coefficient (R) for the same alloy, the improved JC developed by Shokry et al. [48] and the revised FB by Shokry [58] achieve the highest R values of 0.998. Close behind, the revised ZA by Shokry [58], the revised FB models by Li et al. [55] and Fu et al. [57], and the improved JC by Niu et al. [41] give R values of 0.997.

Among the OJC model and its modified versions, the improved version developed by Shokry et al. [48] yields the highest R value and the lowest AARE and RMSE across the four tested alloys. For the FB model and its modifications, the best statistical performance is achieved by the revised version developed by Shokry [58] for all four alloys. When considering the KHL model and its modifications, the improved version developed by Sim et al. [62] provides the best results in terms of R, AARE, and RMSE for the four alloys. Finally, for the ZA model and its modifications, the model revised by Shokry [58] also delivers the optimal values for R, AARE, and RMSE for all four tested alloys.

The proposed modified models in this study effectively incorporate the coupling effects of strain, strain rate, and temperature by introducing a set of interdependent parameters. This enhancement enables the models to accurately reflect the complex, nonlinear behavior observed during hot deformation processes. As a result, these models deliver significantly improved prediction accuracy, particularly under conditions where strong interactions between deformation variables exist. In contrast, models that do not consider these interactions typically perform well only within narrow operating ranges and often fail to predict behavior accurately under more complex conditions. Key advantages of the modified models include their higher accuracy and broader applicability to a range of alloys exhibiting diverse hot flow characteristics. Their strong performance under extreme deformation scenarios also highlights their robustness. Nonetheless, these improvements come with some trade-offs, such as increased computational demands and reduced model simplicity, which may present challenges in practical implementation and interpretation.

Several of the evaluated constitutive frameworks exhibit robust predictive capability, reliably capturing the flow characteristics of the tested alloys with high statistical fidelity. These include the improved version of the JC developed by Shokry et al. [48], the revised versions of FB-based and ZA-based models introduced by Shokry [58], the improved version of the JC presented by Niu et al. [41], the revised version of the FB presented by Li et al. [55], and the revised version of the FB introduced by Fu et al. [57]. Each of these models effectively captures the complex nonlinear behavior of hot deformation by considering numerous correlated parameters related to strain, strain rate, and temperature. Specifically, the model by Shokry et al. [48] incorporates 40 constants, while the models proposed by Shokry [58], Niu et al. [41], Li et al. [78], and Fu et al. [57] include 18, 18, 18, and 16 constants, respectively. However, recent advances in computing power and optimization algorithms, along with user-friendly software environments like MATLAB- and Python-based platforms, have significantly reduced the time and computational cost associated with parameter calibration. Therefore, the time investment required for model fitting has become increasingly negligible in modern industrial and research settings.

In the previous studies, all the modifications studied were applied to specific alloys or element-based alloys, except for the improved version of the JC developed by Shokry et al. [48], which was implemented to investigate various element-based alloys. Consequently, the model demonstrates superior predictive accuracy for the flow characteristics of the various element-based alloys examined in this work. In fact, the other modifications proposed by Shokry [58], Niu et al. [41], Li et al. [78], and Fu et al. [57] also give accurate predictions for the different tested alloys and can be applied for specific alloys. Since these models provide accurate predictions for the four element-based alloys, they are expected to perform well for other alloys with complex hot flow behavior as well. The primary drawback of these modifications is the large number of material constants that need to be accurately determined. However, with significant advancements in computing power and software, this drawback can be overcome.

The inaccurate predictions of hot deformation behavior observed with some of the studied models may be attributed to the following several factors: (1) the models require a large number of material constants that need to be carefully determined, and these constants are not universal, varying significantly with the material; (2) they are typically valid only within a limited temperature range and may not accurately predict deformation behavior outside this range; (3) these models mainly focus on macroscopic behavior, often simplifying microstructural changes during deformation, and fail to account for the complex evolution of grain structure, phase transformations, or other microstructural features that can significantly influence the material’s response; (4) they do not inherently consider frictional effects at the tool–material interface, which can significantly affect material flow during deformation, particularly in extreme strain–rate and high-temperature environments; and (5) in real-world industrial settings, deformation often occurs under complex conditions, such as in multi-pass forging, where temperature gradients, varying strain rates, and complex stress states are present.

## 6. Conclusions

In conclusion, this study demonstrates the application of several phenomenological and physical models, along with their modified versions, to predict flow behavior during hot deformation. These models were specifically applied to four different element-based alloys: nickel-based, aluminum-based, titanium-based, and iron-based. Model precision was substantially increased through parameter identification using the Levenberg–Marquardt method, which iteratively minimizes prediction variance. Statistical parameters correlation coefficient, average absolute relative error, and root mean square error were employed to evaluate the performance of these models comprehensively.

The findings indicate that the modified model based on the JC model, as developed by Shokry et al. [48], demonstrates superior predictive accuracy with an average R value of 0.9985 ± 0.0005, an average AARE of 2.79 ± 1.34%, and an average RMSE of 3.36 ± 1.85 MPa. These values indicate both high accuracy and low variability across the four different alloy systems. Additionally, four other modifications—derived from the FB and ZA models revised by Shokry [58], the JC improved by Niu et al. [41], and the FB models refined by both Li et al. [55] and Fu et al. [57]—also effectively enhance the predictive accuracy for flow behavior. These models achieve an average R value of 0.998 ± 0.0006, an average AARE of 4.28 ± 1.84%, and an average RMSE of 5.53 ± 2.53 MPa across the four different alloy systems. These results underscore the potential of these modifications to improve the modeling of hot deformation processes and provide more precise predictions for various alloys.

In sum, the accuracy of constitutive models is intrinsically linked to the complex interactions between hot working parameters. As the degree of flow behavior becomes more intricate and nonlinear, the number of interactions increases, necessitating a proportional rise in the number of model constants. This expanded set of constants enhances the models’ ability to account for the multifaceted nature of flow behavior, thereby improving the precision of their predictions. However, some models fail to deliver accurate predictions due to inherent limitations, including an insufficient number of constants, the presence of temperature gradients, the neglect of friction effects, and the inability to fully capture the complexity of material behavior. These challenges highlight the importance of developing more comprehensive models that effectively address these factors to achieve higher predictive accuracy.

Such an extension would significantly contribute to the development of advanced materials by facilitating the design of alloys with tailored mechanical properties and enhanced performance. Moreover, applying these models to new materials would support efforts to improve the service life of critical components used in demanding applications. Additionally, by accurately modeling the flow behavior, the proposed approach can aid in optimizing hot deformation parameters, which in turn can lead to reduced energy consumption, minimized manufacturing defects, and overall improved efficiency in industrial processing. The authors consider this a promising direction for future research and intend to explore it in subsequent studies.

## Data Availability

Data will be available upon request through the corresponding author.
